# Posterior communicating artery vasospasm impairs cerebral pulsatility in an experimental subarachnoid hemorrhage model

**DOI:** 10.3389/fneur.2025.1649577

**Published:** 2025-10-08

**Authors:** İskender Samet Daltaban, Mehmet Selim Gel, Ayhan Kanat, Mehmet Dumlu Aydın, Songül Turğut

**Affiliations:** ^1^Department of Neurosurgery, Alife Hospital, Ankara, Türkiye; ^2^Department of Neurosurgery, Faculty of Medicine, Trabzon University, Trabzon, Türkiye; ^3^Department of Neurosurgery, Medical Faculty, Recep Tayyip Erdogan University, Rize, Türkiye; ^4^Department of Neurosurgery, Faculty of Medicine, Atatürk University, Erzurum, Türkiye; ^5^Department of Neurology, Medicana Interantional Ankara Hospital, Ankara, Türkiye

**Keywords:** subarachnoid hemorrhage, cerebral vasospasm, posterior cerebral artery, intracranial pressure, animal experimentation

## Abstract

**Background:**

Subarachnoid hemorrhage (SAH) commonly causes cerebral vasospasm and delayed ischemia. Spasm of the posterior communicating artery (PCoA) can disrupt cerebral hemodynamics. We assessed its effect on cerebral pulsatility in a rabbit SAH model, focusing on intracranial pressure pulse amplitude as an early, sensitive marker of vasospasm-driven change, whereas global CBF or neurological outcomes generally require larger or longer studies.

**Methods:**

Rabbits were randomly assigned to three groups: control (no injection), sham-controlled (saline injection), and SAH (0.75 mL autologous blood injection into the basal subarachnoid space near the PCoA under anesthesia). Heart rate and cerebral pulsation amplitude (measured via intracranial pressure transducer) were recorded on day 1 and day 7. On day 7, animals were euthanized, and histological analysis of the PCoA was performed. Vasospasm index (VSI) was calculated as the ratio of arterial wall area to lumen area. Group comparisons and temporal changes were assessed statistically.

**Results:**

Twenty-three rabbits completed the study (five control, five sham-controlled, 13 SAH, two SAH animals excluded due to early mortality). On day 1, the SAH group showed a significantly reduced pulsation amplitude compared to controls and sham-controlled groups. By day 7, pulsation amplitude partially recovered in the SAH group but remained lower than in controls. Control and sham-controlled groups exhibited a slight, non-significant decline in pulsation. The VSI was highest in the SAH group, moderate in sham-controlled, and lowest in the control groups. Heart rate declined over time across all groups, with significant bradycardia in the SAH group by day 7.

**Conclusion:**

PCoA vasospasm following experimental SAH results in a sustained reduction in cerebral pulsatility. These findings suggest that localized vasospasm disrupts pulsatile intracranial dynamics, potentially contributing to SAH-related pathophysiology.

## Introduction

Aneurysmal subarachnoid hemorrhage (SAH) is a life-threatening emergency frequently complicated by cerebral vasospasm and delayed cerebral ischemia and carries high morbidity and mortality, in part due to cerebral vasospasm that often follows the initial hemorrhage (1, 2). Cerebral vasospasm is a prolonged, reversible narrowing of the cerebral arteries that typically occurs a few days after SAH and can lead to delayed cerebral ischemia (1, 3). It has been documented that vasospasm contributes significantly to poor outcomes after SAH (3–5). Standard monitoring of vasospasm in patients relies on angiography or transcranial Doppler (TCD) ultrasound to detect elevated blood flow velocities (6, 7). However, vasospasm may also alter cerebral pulsatility, the pulsatile component of cerebral blood flow or pressure, which is not routinely assessed (7, 8).

**Figure 1:**
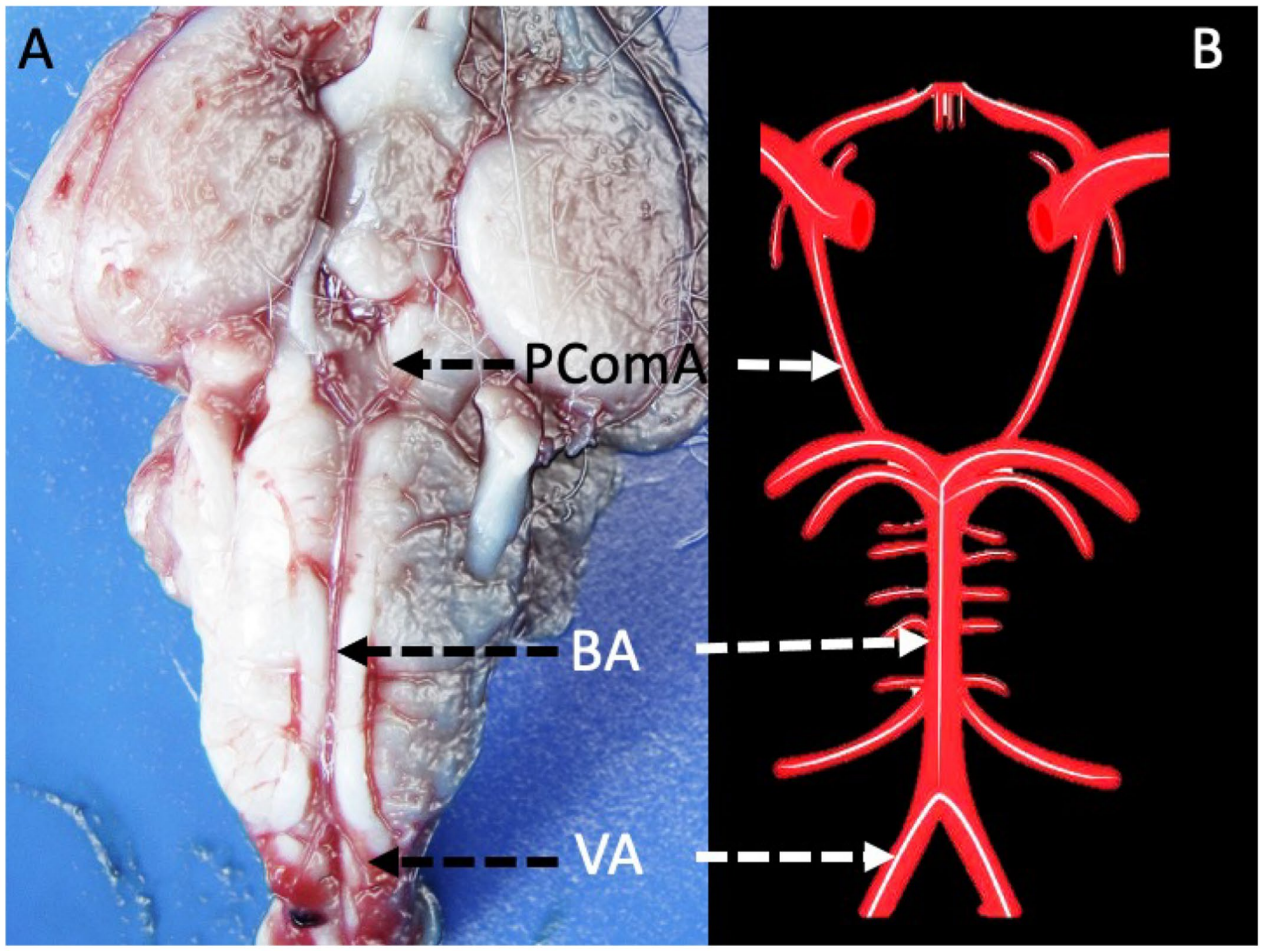
**(A,B)** Illustrate the anatomical configuration of the basal cerebral arterial system in a healthy brain, highlighting key vessels including the posterior communicating artery (PComA), basilar artery (BA), and vertebral arteries (VA).

The posterior communicating artery (PCoA) serves as a crucial collateral pathway between the anterior and posterior cerebral circulations (Figure 1). In the event of arterial occlusion or stenosis, a functional PCoA can redirect blood flow to maintain cerebral perfusion (9). However, vasospasm of the PCoA can hinder this compensatory mechanism, increasing the risk of ischemia. Due to its location in the basal cisterns, the PCoA is often exposed to subarachnoid blood following aneurysm rupture, making it susceptible to vasospasm. Additionally, its proximity to the oculomotor nerve means that aneurysms or severe spasms in the PCoA can compress or cause ischemic injury to this nerve, leading to cranial nerve III palsy (10). Experimental studies have demonstrated that SAH can result in various neurovascular dysfunctions, including cranial nerve injuries and autonomic disturbances, even in the absence of direct brain ischemia. Takahashi et al. (11) reported that acute SAH can trigger hypothalamic dysfunction and a catecholamine surge, leading to arrhythmias, cardiac stunning, and pulmonary edema.

Cerebral pulsatility refers to the rhythmic variations in cerebral blood volume and pressure with each cardiac cycle. It can be quantified using metrics like the pulsatility index (PI) on TCD, which is derived from arterial blood flow velocity waveforms, or by measuring the amplitude of intracranial pressure pulsations (12). Cerebral pulsatility is influenced by arterial stiffness, cerebrovascular resistance, and intracranial compliance. Changes in pulsatility may provide insight into the cerebrovascular condition: for instance, an elevated PI can indicate increased distal resistance or decreased compliance, whereas a very low PI can indicate vasodilation or loss of tone (6, 12, 13). Rajajee et al. (6) demonstrated that a low PI measured by transcranial Doppler within the first 48 h after aneurysmal SAH independently predicted subsequent large-vessel vasospasm. In clinical SAH, pulsatility changes have been observed, some studies report that an abnormally low PI in the early phase after SAH is associated with subsequent development of large-vessel vasospasm, possibly reflecting initial dysregulation of cerebrovascular tone before vasospasm onsets. On the other hand, as vasospasm progresses and distal resistance rises, pulsatility might increase (14). Despite these observations, the direct relationship between a specific artery’s vasospasm and cerebral pulsatility remains incompletely understood.

In this context, we conducted an experimental study to investigate the specific impact of PCoA vasospasm on cerebral pulsatility. Employing a rabbit model of SAH, we induced focal vasospasm in the PCoA and simultaneously monitored intracranial pressure dynamics to quantify pulsatile flow characteristics. Our hypothesis was that severe constriction of the PCoA, by compromising collateral pathways within the circle of Willis, would attenuate the normal transmission of arterial pulse waves into the cranial compartment, thereby reducing intracranial pressure pulsation amplitude. To isolate this effect, we compared animals subjected to SAH-induced vasospasm with sham-controlled and control groups, ensuring that observed differences reflected arterial narrowing rather than procedural or systemic influences. Cerebral pulsatility, assessed through beat-to-beat ICP pulse amplitude, represents an integrative parameter influenced by large-vessel stiffness, distal cerebrovascular resistance, and intracranial compliance. Thus, early reductions in amplitude may signal impaired hemodynamic transmission during acute SAH, while subsequent alterations in pulsatility indices may correspond to the evolution of vasospasm. Unlike global cerebral blood flow or neurological function, which require larger cohorts and longer follow-up for accurate characterization, ICP pulsatility offers a sensitive and immediate hemodynamic marker. We therefore postulated that SAH-induced PCoA vasospasm would acutely depress ICP pulsatility with only partial recovery by day seven, in parallel with histologically verified vasospasm severity. This approach aims to improve our understanding of the pathophysiological consequences of localized vasospasm and to highlight pulsatility metrics as potential adjunctive parameters for monitoring SAH patients beyond conventional flow velocity assessments.

## Materials and methods

### Experimental design and animal groups

Twenty-five male rabbits aged 3 ± 0.4 years were included in this experiment. Animals were divided into three groups: control (*n* = 5), Sham-controlled (*n* = 5), and SAH study group (*n* = 15). Animal husbandry and study protocols adhered strictly to the guidelines of the National Institutes of Health and were approved by the Committee on Animal Research of Atatürk University.

### Anesthesia and euthanasia protocol

All procedures were performed under sterile conditions with rabbits under general anesthesia. Premedication consisted of acepromazine (1 mg/kg IM) and xylazine (5 mg/kg IM), followed by induction with ketamine hydrochloride (25–35 mg/kg IM). In some animals, lidocaine hydrochloride (15 mg/kg IM) was also administered during induction, and additional doses were given as necessary to maintain surgical anesthesia. General anesthesia was maintained using inhaled isoflurane (1–2%) in 100% oxygen. Adequacy of anesthesia was confirmed by the absence of withdrawal reflexes. Rabbits were positioned prone, and the occipitocervical region was prepared with antiseptic solution prior to surgical procedures.

Postoperative analgesia was provided using buprenorphine (0.05 mg/kg SC every 12 h for 48 h). At the experimental endpoint (day 14), animals were euthanized by intravenous administration of pentobarbital (150 mg/kg).

### Induction of subarachnoid hemorrhage and vasospasm

In the SAH group, subarachnoid hemorrhage was induced by injecting 0.75–1.5 mL of autologous arterial blood into the cisterna magna. Autologous blood was drawn immediately prior to injection from the auricular artery. A 22-gauge needle was carefully inserted through the atlanto-occipital membrane, allowing slow injection into the cerebrospinal fluid space, targeting distribution around the circle of Willis, thus reliably inducing vasospasm. After injection, the needle was withdrawn, and the puncture site sealed with tissue adhesive. Rabbits were positioned in a head-down tilt for approximately 10 min post-injection to facilitate blood pooling around the basal arteries and subsequently returned to horizontal position for recovery.

Sham-controlled group rabbits underwent the identical procedure but received an injection of 0.75–1.5 mL sterile saline instead of blood, controlling for mechanical impact and acute intracranial pressure changes without the spasmogenic factor of blood. Control group rabbits underwent anesthesia and needle placement without any injection to account for anesthesia-related effects.

Post-procedurally, rabbits received subcutaneous buprenorphine (0.05 mg/kg) for analgesia and were closely monitored during recovery and throughout the 7-day follow-up period. Standard laboratory care was provided, with animals observed for neurological impairments. Any rabbit showing severe neurological deterioration (e.g., coma and respiratory failure) was euthanized humanely. Two rabbits from the SAH group succumbed to immediate complications from the procedure and were excluded from the final analysis, leaving 13 animals completing the study in the SAH group.

Prior to euthanasia, electrocardiographic monitoring was conducted to record heart rhythms. Additionally, a right parietal burr-hole was drilled using a perforator, and cerebral pulsations per minute were counted and recorded using portable ultrasound. Following euthanasia, rabbit brains were fixed in 10% formalin for 7 days. Sections (5 μm) of the basilar arteries at mid-pontine levels were stained with hematoxylin and eosin for histological examination. Vasospasm assessment was quantified as the wall surface/lumen surface ratio, calculated by subtracting the lumen surface from the total arterial wall surface.

### Measurement of cerebral pulsatility and physiological parameters

We evaluated cerebral pulsatility by measuring the pulsatile component of intracranial pressure (ICP) in all groups on day 1 (24 h after SAH) and on day 7 (the endpoint) in the guide of previous studies (15, 16). Under general anesthesia, a small burr hole was created in the right parietal bone, and a Codman^®^ intracranial pressure (ICP) microsensor probe (Johnson & Johnson, Codman Division, Randolph, MA, United States) was placed epidurally or in the subdural space adjacent to the cortex. The probe was calibrated prior to each use according to the manufacturer’s instructions. ICP waveforms were continuously recorded using a standard bedside monitor, and the analog trace was stored for offline analysis. Brain pulsation amplitude was defined as the peak-to-trough difference of the cardiac-synchronous ICP wave, representing the magnitude of intracranial pulsatility in mmHg. Importantly, no ultrasound techniques were employed for pulsatility measurement; all data were derived directly from the ICP transducer waveform. This ensured that the parameter termed “brain pulsation” in the present study corresponded to a validated and widely accepted method of direct ICP monitoring rather than indirect surrogate measures. To record ICP, rabbits were re-anesthetized (with the same ketamine/xylazine regimen) at the specified time points. Under aseptic conditions, a small burr hole was made in the right parietal skull (approximately 3 mm diameter). A pressure transducer (intracranial pressure monitor, Codman ICP Microsensor or equivalent) was inserted epidurally just beneath the bone into the subdural space to detect pulsatile pressure waves. Care was taken to avoid significant bleeding or brain injury during placement. The transducer was connected to a patient monitor for pressure waveform recording. After an equilibration period, continuous ICP was recorded for 5 min. From this recording, the brain pulsation amplitude was calculated as the mean pulse pressure (the difference between systolic and diastolic ICP) over the recording interval. This pulsation amplitude (in mmHg or mmH₂O) reflects the magnitude of intracranial pressure change with each heartbeat, serving as an index of cerebral pulsatility.

During the same monitoring periods, heart rate (HR) was recorded using lead II electrocardiography. Heart rate provides a general physiologic status and ensures that any differences in pulsatility are not simply due to cardiac rate changes. Body temperature was maintained with a warming pad during anesthesia to prevent hypothermia (which could affect cardiovascular measures). In addition, general observations such as level of consciousness and limb movements were noted daily to qualitatively assess neurologic status.

After recording measurements on day 1, the burr hole was sealed with bone wax and the scalp sutured to allow the rabbit to recover until day 7. On day 7, the ICP pulsatility measurement was repeated in the same manner. Following the final measurements, the animals were deeply anesthetized and then perfused transcardially with saline followed by 10% buffered formalin for tissue fixation. We followed the saline injection dosages as reported in the study of Kamp et al. (17). In the SAH group, a fixed volume of 0.75 mL of autologous arterial blood was injected into the basal subarachnoid cistern through the cisterna magna. Although preliminary pilot experiments tested volumes up to 1.5 mL, the final experimental protocol standardized the injection to 0.75 mL in all animals to ensure reproducibility and avoid variability. This volume was selected on the basis of prior studies demonstrating that approximately 0.75 mL reliably induces subarachnoid hemorrhage and vasospasm in rabbits of comparable size (3–3.5 kg), without excessive mortality risk (18). Importantly, the injection volume was not scaled to body weight but kept constant across animals, corresponding to roughly 10% of the rabbit’s circulating blood volume, a proportion commonly employed in experimental SAH models to achieve consistent hemorrhage induction. Standardization to 0.75 mL thus provided uniformity across experimental subjects and eliminated potential confounding introduced by a variable injection range. In contrast to rat models where smaller volumes (e.g., 0.2 mL) are sufficient to induce SAH due to the limited subarachnoid space, rabbits require larger injection volumes. Based on previous rabbit studies and considering the greater circulating and cerebrospinal volume of New Zealand male rabbits (~3–3.5 kg), a fixed dose of 0.75 mL autologous arterial blood was chosen to ensure reproducible induction of SAH and vasospasm while maintaining acceptable survival rates (19).

### Histopathological assessment of vasospasm

Immediately after euthanasia on day 7, the brain was carefully extracted with the circle of Willis intact. The PCoAs from both sides were identified under a surgical microscope. For consistency, we focused on the right PCoA (or the more accessible side) for quantitative analysis. The PCoA was dissected free along with adjacent brain tissue and further fixed in formalin for at least 24 h. Fixed specimens were then embedded in paraffin. Standard histological sections (5 μm thick) were taken perpendicular to the long axis of the PCoA at the mid-portion of the artery. Sections were stained with hematoxylin and eosin (H&E) for general morphology. In addition, Verhoeff-Van Gieson elastic stain was applied on adjacent sections to delineate the internal elastic lamina and overall arterial wall structure in some cases.

**Figure 2:**
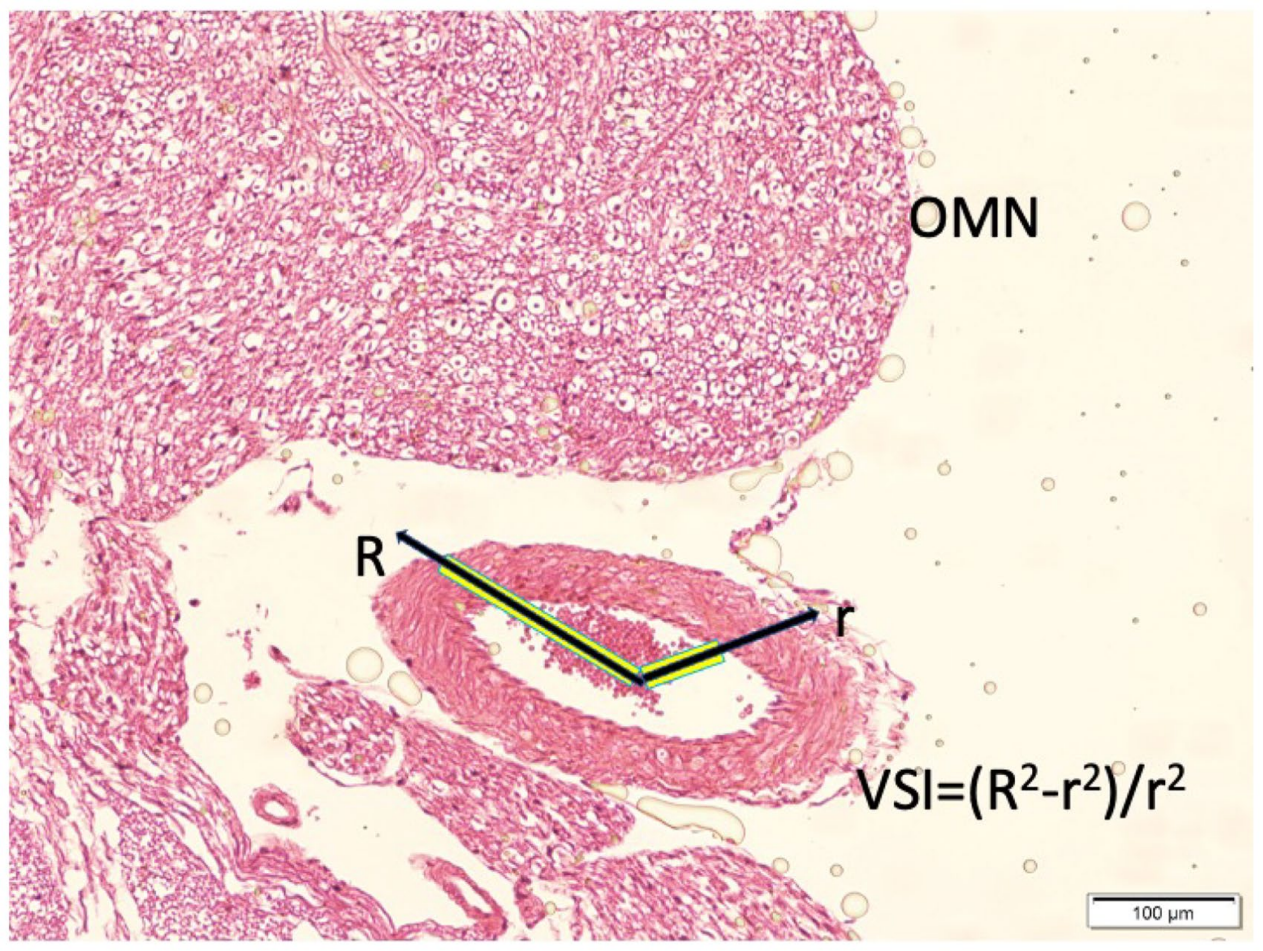
Histopathological representation of the posterior communicating artery (PComA) demonstrating the methodology for calculating the vasospasm index (VSI). The cross-sectional area of the arterial wall and lumen are delineated for morphometric analysis. The VSI is defined as the ratio of wall area to lumen area, reflecting the degree of vasospasm severity (light microscopy, hematoxylin and eosin, ×10).

Histological sections were examined by light microscopy. Vasospasm severity was quantified using a vasospasm index (VSI), defined as the ratio of the arterial wall area to the lumen area (Figure 2). To calculate this, digital micrographs of the PCoA cross-sections (at 100× magnification) were analyzed with image analysis software. The lumen area (the area within the endothelium) and the total arterial area (outer boundary of the media/adventitia) were measured. The arterial wall area was obtained by subtracting the lumen area from the total arterial area. For each animal, three non-overlapping cross-sections were measured and the results averaged. The VSI = wall area/lumen area is a dimensionless ratio: a higher VSI indicates a relatively thick arterial wall and narrow lumen, consistent with vasospasm. A normal artery with wide lumen would have a low VSI. This index provides a quantitative measure of vasospasm that is less sensitive to absolute artery size and more reflective of narrowing. Direct measurements of wall thickness were performed at three to four equidistant points along the circumference, and the mean value was calculated for each vessel. This dual approach ensured that irregular or elliptical vessel shapes were accurately represented in the morphometric analysis. Additionally, qualitative features of vasospasm were noted, including corrugation of the internal elastic membrane, thickness of the smooth muscle layer, and any signs of endothelial damage or perivascular inflammatory cells.

### Outcome measures and statistical analysis

The primary outcome measures were cerebral pulsation amplitude and the posterior communicating artery (PCoA) vasospasm index (VSI), while heart rate changes over time were analyzed as secondary outcomes. Data were summarized as mean ± standard deviation. Given the relatively small sample sizes and the non-normal distribution of several variables, all analyses were conducted using non-parametric statistical methods. Overall group differences for continuous variables were first assessed using the Kruskal–Wallis test. When this analysis indicated a statistically significant difference, Dunn’s multiple comparison *post hoc* test with Bonferroni correction was applied to identify significant pairwise differences among the three groups (SAH vs. control, SAH vs. sham, and sham vs. control). Within-group changes between day 1 and day 7 were evaluated using the Wilcoxon signed-rank test for paired non-parametric data. A two-tailed *p*-value <0.05 was considered statistically significant for all analyses. To facilitate interpretation, exact *p*-values for key comparisons are reported in the Results. Statistical analyses were performed using SPSS version 26.0 (IBM Corp., Armonk, NY, United States).

## Results

### General observations

All control and sham-controlled group rabbits remained neurologically normal throughout the 7-day observation period. In the SAH group, the induction of subarachnoid hemorrhage produced transient neurological disturbances such as brief loss of consciousness and sluggishness in activity during the first 24–48 h. Two rabbits in the SAH group died within the first 48 h post-SAH (mortality 13.3% for SAH group), likely due to the severity of the hemorrhage or acute increased intracranial pressure; these were excluded from analysis. The remaining SAH rabbits survived to the end of the experiment, though they exhibited mild neurologic deficits (e.g., reduced appetite and slight imbalance) compared to controls. No sham-controlled or control animals died or showed neurological deficits, indicating that the surgical procedure and saline injection alone did not cause significant harm.

### Intracranial pulsatility changes

**Figure 3:**
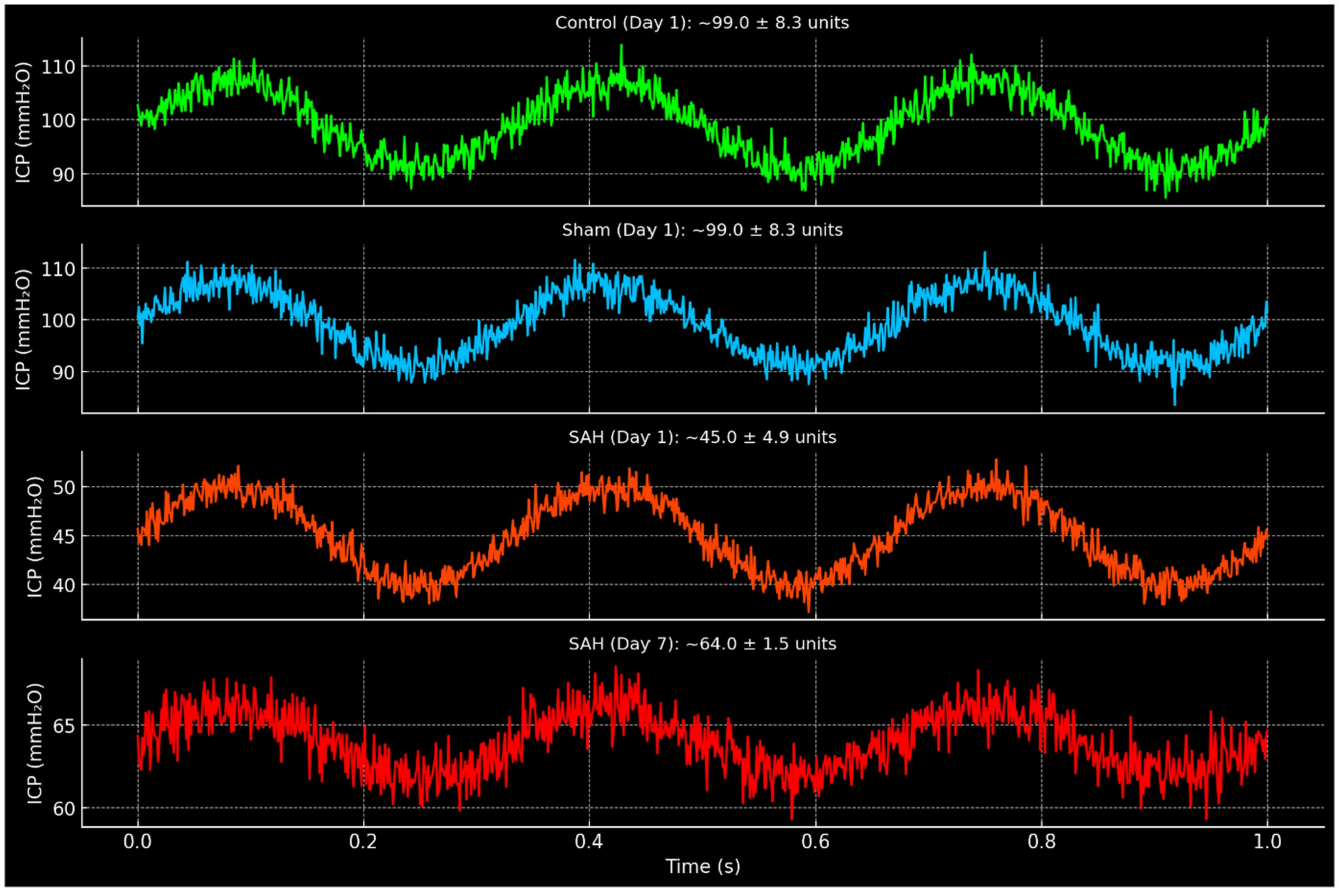
Representative intracranial pressure (ICP) waveforms in control, sham, and SAH groups. Waveforms are displayed in mmH₂O units and annotated with corresponding mean ± SD values derived from experimental data. Control and sham groups at day 1 demonstrated normal cardiac-synchronous pulsations (~99.0 ± 8.3 units). SAH animals exhibited markedly reduced amplitude on day 1 (~45.0 ± 4.9 units). By day 7, SAH animals showed partial recovery (~64.0 ± 1.5 units), though amplitudes remained lower than control and sham values at the same time point (~83 units).

On day 1 (24 h after hemorrhage), there was a clear difference in brain pulsation amplitude between groups. The SAH group had a dramatically lower intracranial pulsation amplitude compared to both control and sham-controlled groups (*p* < 0.01 for each comparison). In control rabbits, the mean pulsation amplitude at day 1 was 99.0 ± 8.3 units (arbitrary units corresponding to mm H₂O of ICP pulse, see Table 1 and Figure 3), and in sham-controlled rabbits it was 99.0 ± 8.3, essentially identical to controls on average. In contrast, SAH rabbits on day 1 showed a mean pulsation amplitude of only 45.0 ± 4.9 units—less than half that of the controls (Table 1). This marked reduction in pulsatility in the SAH group indicates that the presence of subarachnoid blood and the incipient vasospasm had an acute dampening effect on the brain’s pulsatile pressure dynamics. Notably, there was no significant difference between the control and sham-controlled groups’ pulsatility on day 1, implying that the saline injection itself did not significantly alter intracranial pulsations in the acute phase.

**Table 1:** Summary of heart rate, brain pulsation amplitude, and vasospasm index in each group.

Group	Heart rate day 1 (bpm)	Heart rate day 7 (bpm)	Brain pulsation day 1 (units)	Brain pulsation day 7 (units)	Vasospasm index (day 7)
Control	218 ± 17	203 ± 10	99.0 ± 8.3	83.0 ± 10.0	0.40 ± 0.07
Sham-controlled	206 ± 14	187 ± 20	99.0 ± 8.3	83.0 ± 6.7	0.99 ± 0.10^*^
SAH	156 ± 9^*,†^	98 ± 5^*,†^	45.0 ± 4.9^*,†^	64.0 ± 1.5^*,†,‡^	1.86 ± 0.21^*,†^

Data are presented as mean ± SD. Group differences were analyzed using Kruskal–Wallis with Dunn’s *post hoc* test (Bonferroni corrected). ^*^p < 0.05 vs. control; ^†^*p* < 0.01 vs. sham-controlled; ^‡^*p* < 0.01 vs. control.

Between day 1 and day 7, temporal changes in pulsatility showed opposite trends for SAH vs. non-SAH animals. In the SAH group, the cerebral pulsation amplitude increased substantially over time. By day 7, the mean pulsation amplitude in SAH rabbits rose to 64.0 ± 1.5 units. This represented a significant within-group increase compared to day 1 (*p* < 0.01, Wilcoxon test). In fact, every surviving SAH animal showed a higher pulsation amplitude on day 7 than on day 1. In contrast, the control group’s pulsation amplitude decreased slightly by day 7 (mean 83.0 ± 10.0, down from 99.0; not statistically significant, *p* = 0.07), and similarly the sham-controlled group’s amplitude decreased to 83.0 ± 6.7 (from 99.0; not significant). The small declines in control and sham-controlled groups may reflect a normal adaptation or the effects of a week’s aging or repeated anesthesia, but importantly they were minor. Thus, the SAH group exhibited a unique dynamic: an initially depressed pulsatility that partially rebounded over the course of a week.

Despite the rebound, on day 7 the SAH group’s pulsation amplitude was still lower than that of the control group. The mean amplitude in SAH (64.0) remained about 23% lower than in controls (83.0). This difference was statistically significant (*p* < 0.05). The sham-controlled group’s amplitude (83.0) was virtually identical to controls on day 7, and significantly higher than the SAH group (*p* < 0.05). These data indicate that even after a week, with vasospasm fully developed, the SAH animals did not restore normal pulsatile dynamics; their intracranial pulsatility remained impaired relative to baseline normal animals.

To summarize the pulsatility findings (illustrated in Table 1), SAH induced a biphasic effect on cerebral pulsations: an acute suppression followed by a partial recovery, but not to normal levels. Non-SAH animals maintained pulsatility near baseline or with a mild decline. The presence of subarachnoid blood and subsequent vasospasm in the SAH group was associated with persistent alteration of the normal intracranial pulse pressure pattern.

### Heart rate and clinical parameters

All groups had comparable heart rates at baseline (prior to any injection, during induction of anesthesia). On day 1 after the procedure, the measured heart rates in anesthetized rabbits were not significantly different among groups: the control group had an average HR of 218 ± 17 beats per minute (bpm), sham-controlled group 206 ± 14 bpm, and SAH group 156 ± 9 bpm (see Table 1). Although the SAH group’s mean appeared lower, it is important to note that these HR values were obtained under anesthesia at 24 h post-insult, and some of the difference may be due to the physiological stress of SAH. By day 7, more pronounced differences emerged. The control and sham-controlled groups still had relatively high heart rates under anesthesia (around 203 ± 10 bpm for control and 187 ± 20 bpm for sham-controlled), whereas the SAH group’s mean heart rate had further decreased to 98 ± 5 bpm. This bradycardia in the SAH group by day 7 was significant (*p* < 0.01 vs. control; *p* < 0.05 vs. sham-controlled controlled). In conscious daily observations, SAH animals also had a trend toward lower resting heart rates and occasional episodes of bradycardia. The development of bradycardia in the SAH group could be related to Cushing’s response (as a reaction to increased intracranial pressure) or brainstem involvement due to the SAH and vasospasm near vital centers. In contrast, control and sham-controlled animals maintained normal heart rates. Importantly, heart rate changes did not artifactually cause the pulsatility changes, a lower heart rate typically allows more time for pulse pressure equilibration and could reduce pulsatility slightly, but the dramatic difference in pulsation amplitude is more directly attributable to the vascular factors rather than heart rate.

No significant changes in systemic arterial blood pressure were noted (blood pressure was not invasively monitored continuously, but intermittent measurements showed no hypotension in any group). There were no signs of infection or fever in any group, and body weights remained stable over 7 days (with slight loss in SAH group likely due to reduced feeding).

### Vasospasm index and histological findings

**Figure 4:**
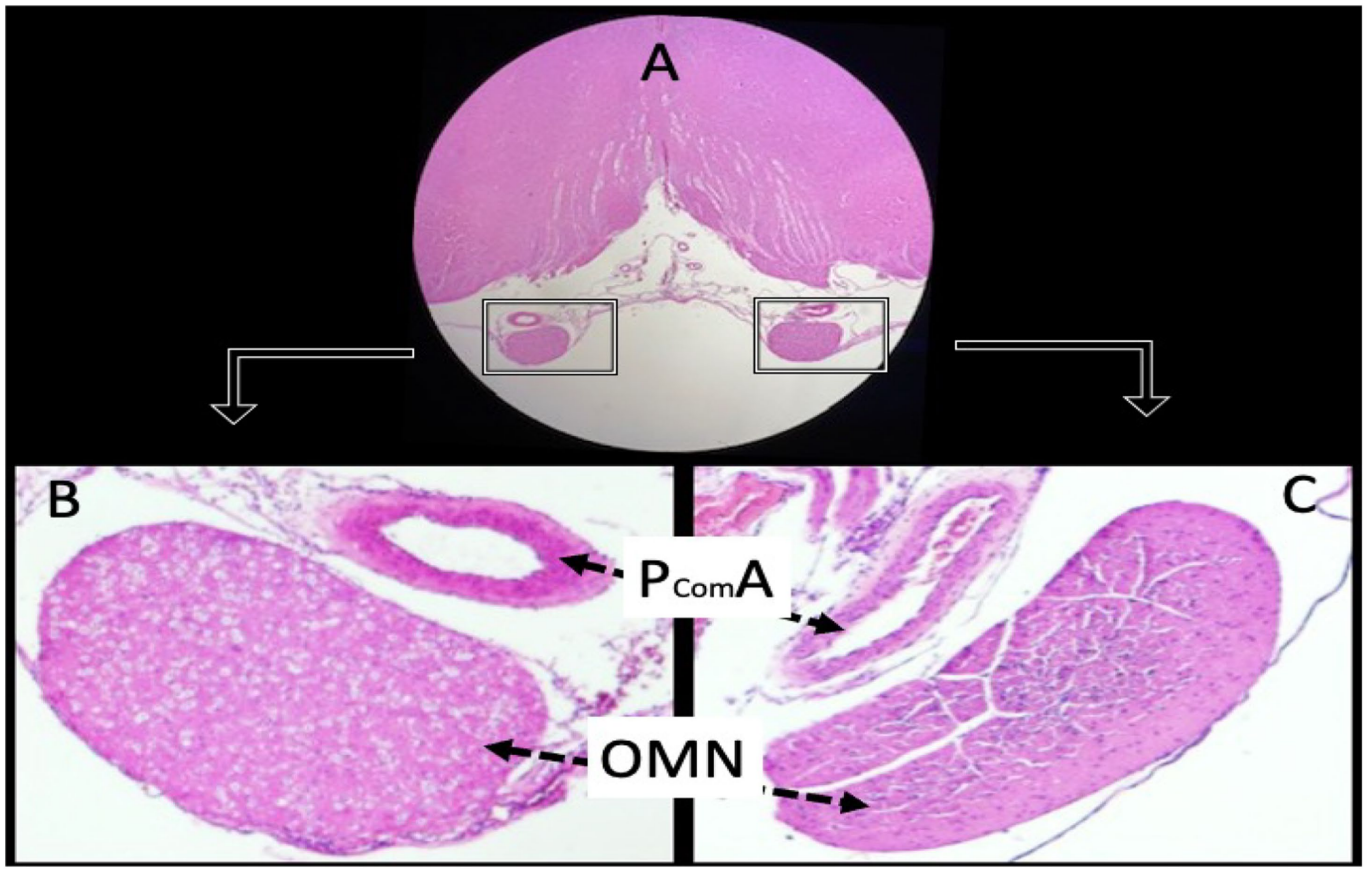
Histological sections of a normal animal demonstrate the anatomical relationship between the oculomotor nerves (OMN) and the posterior communicating arteries (PComA). Panel **(A)** shows the OMN under low magnification (hematoxylin and eosin, ×4). Panels **(B,C)** depict the PComA with preserved luminal architecture and intact vascular wall morphology (light microscopy, H&E, ×4).

Histopathological examination of the PCoA on day 7 revealed clear differences between groups, consistent with the presence or absence of vasospasm. In control rabbits, the PCoA cross-sections showed a wide, patent lumen and thin vessel walls with normal histology (Figure 4). The endothelial layer was intact, and the internal elastic lamina appeared as a smooth, wavy line. There were no signs of vessel wall thickening or narrowing.

**Figure 5:**
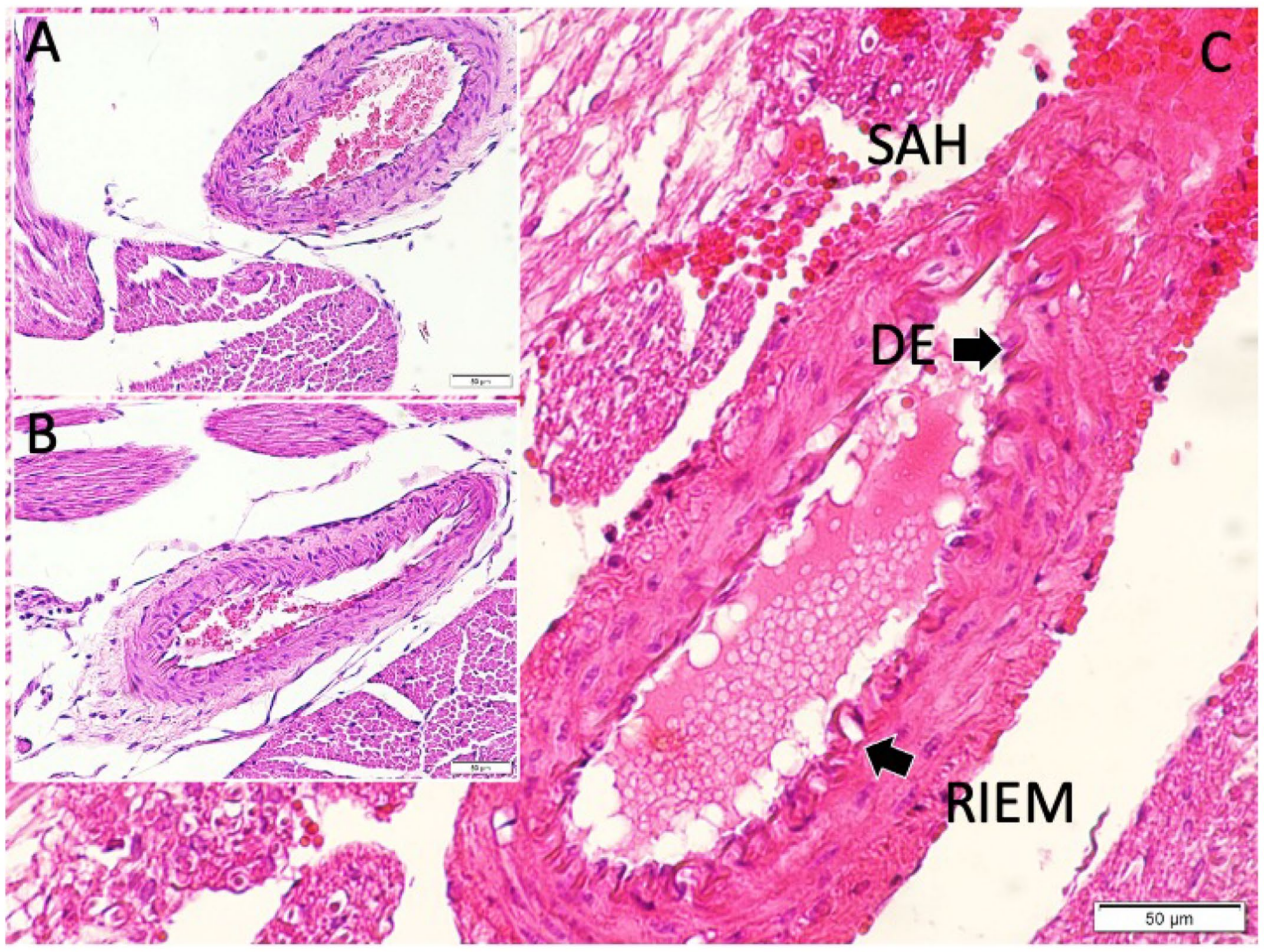
Histological examination of the posterior communicating artery revealed clear intergroup differences. In the control animals, the lumen was widely patent with thin vessel walls, while sham animals exhibited mild narrowing and modest wall thickening. In contrast, SAH animals showed marked luminal constriction and pronounced wall hypertrophy. Panel **(A)** illustrates the normal arterial architecture in the control group, with a wide lumen and thin vessel wall. Panel **(B)** shows a PComA from a sham-controlled animal, exhibiting mild wall thickening and subtle luminal narrowing. Panel **(C)** displays a PComA from a study (SAH) animal, demonstrating marked luminal constriction and prominent vascular wall thickening indicative of severe vasospasm (light microscopy, hematoxylin and eosin, ×20). DE, disrupted endothelium: This label in the figure points to areas where the endothelial layer appears discontinuous or damaged in the vasospastic artery (SAH group). We chose “DE” to mark regions of endothelial cell loss or irregularity, which are commonly observed in vasospastic arteries (endothelial damage is part of the vasospasm pathology). RIEM, redundant internal elastic membrane: This term refers to the wavy, folded appearance of the internal elastic lamina of the artery under vasospasm. In severe vasospasm, because the vessel constricts, the internal elastic membrane (IEM) often appears corrugated or “redundant.” We used the abbreviation “RIEM” to indicate redundant (or folded) IEM. In the figure, arrows labeled RIEM point to the scalloped elastic lamina in the vessel wall of the SAH group’s PCoA section (seen with H&E stain as undulating lines). This feature contrasts with the relatively straight IEM in the non-vasospastic control.

Sham-controlled rabbits (saline-injected) exhibited mild vasospasm changes: the PCoA lumen was somewhat smaller than controls and the arterial wall slightly thicker (Figure 5). Some corrugation of the internal elastic membrane and a mild degree of subendothelial edema were also observed. These findings suggest that even saline injection can provoke a minor irritative vasospastic response, likely due to a transient increase in intracranial pressure or mechanical stretch.

The SAH group rabbits showed pronounced vasospasm in the PCoA. The luminal area was greatly reduced, and the vessel walls were markedly thickened (Figure 5C). The internal elastic lamina appeared folded and convoluted, indicative of vessel constriction. The smooth muscle layer was hypertrophied. In some sections, vacuolar changes and perivascular inflammatory cell infiltrates—likely neutrophils or macrophages—were noted within the adventitia. Endothelial cell irregularities, such as areas of denudation, were also present, possibly due to the cytotoxic effect of subarachnoid blood. These qualitative features confirm that the blood injection led to severe arterial narrowing and structural changes characteristic of vasospasm.

Quantitatively, VSI differed significantly among groups (Table 1). Control rabbits had a mean VSI of 0.40 ± 0.07. Sham-controlled rabbits had a mean of 0.99 ± 0.10, which was more than double the control value, reflecting a moderate degree of narrowing from saline injection. The SAH group’s mean VSI was 1.86 ± 0.21, approximately 4.5 times higher than control. Statistical analysis showed a significant overall group difference (*p* < 0.001, Kruskal–Wallis). Pairwise comparisons confirmed that SAH VSI was significantly higher than both sham-controlled and control groups (*p* < 0.0001 vs. control; *p* < 0.001 vs. sham). The sham-controlled group’s VSI was also significantly higher than control (*p* < 0.01), indicating a mild but notable vasospasm effect from saline alone (individual animal VSI values are provided in Supplementary Table 1).

These VSI measurements quantitatively validate the histological impressions: SAH induces the most severe vasospasm, sham induces mild vasospasm, and controls have none. The consistency of high VSI in all SAH animals suggests that our model reliably produced vasospasm in the PCoA by day 7 in surviving subjects. We did not find a significant side-to-side difference (left vs. right PCoA); vasospasm tended to be bilateral, although measurements were focused on one side for consistency.

Lastly, we examined whether VSI correlated with changes in pulsation amplitude among SAH animals. An inverse trend was noted: animals with the highest VSI tended to have the lowest pulsation amplitudes on day 7, whereas those with slightly lower VSI had somewhat higher pulsation. Due to the relatively small sample, this correlation did not reach statistical significance (Spearman *ρ* = −0.45, *p* = 0.12). Nonetheless, the trend supports the notion that more severe PCoA spasm impairs cerebral pulsatility to a greater extent. Using an area-based method, VSI values were recalculated for all groups, yielding results consistent with the primary analysis (control: 0.38 ± 0.07; sham: 1.00 ± 0.10; SAH: 1.90 ± 0.21).

## Discussion

This experimental study demonstrates that vasospasm of the posterior communicating artery can significantly impair cerebral pulsatility following subarachnoid hemorrhage. Using a rabbit SAH model, we observed that animals with PCoA vasospasm developed a markedly altered intracranial pressure pulsation profile. Specifically, the SAH-induced vasospasm led to an acute reduction in the amplitude of intracranial pulsations, followed by a partial restoration as the vasospasm became chronic, but pulsatility remained subnormal by 1 week. In parallel, the PCoA in these animals showed clear pathological evidence of vasospasm, with lumen narrowing and vessel wall thickening. In contrast, control and sham-controlled animals without significant vasospasm maintained normal pulsatility throughout. To our knowledge, this is the first study focusing on how isolated arterial vasospasm (here, of the PCoA) affects the pulsatile dynamics of the intracranial compartment in an experimental setting.

Our findings contribute to the understanding of SAH pathophysiology by linking macroscopic vascular changes (arterial narrowing) with intracranial hemodynamic consequences (pulsatility changes). Cerebral vasospasm after SAH is classically known to reduce vessel diameter and cerebral blood flow, potentially leading to ischemia in brain tissue (20, 21). However, even before explicit ischemia occurs, vasospastic constriction can alter the characteristics of blood flow and pressure wave transmission (22). In the SAH group, we observed that 24 h post-hemorrhage the intracranial pulsation amplitude dropped to about half of normal. One plausible explanation for this early dampening of pulsatility is the effect of acute SAH on intracranial pressure and vascular tone. Additionally, acute SAH is known to trigger a catecholamine surge and early vasomotor dysfunction; some arteries may initially dilate or lose tone before the development of delayed vasospasm, particularly if transient hypertension or autoregulatory breakthrough occurs (6, 11). The introduction of blood into the subarachnoid space acutely raises intracranial pressure (ICP); although we did not continuously monitor ICP, the acute phase of our model likely involved a transient elevation of baseline ICP. An elevated baseline ICP can compress the intracranial vasculature and reduce the compliance of the intracranial compartment, paradoxically leading to a blunting of pulsatile fluctuations if the intracranial system is pushed toward the nonlinear compliance curve plateau. In simple terms, when the brain is acutely swollen or overfilled with blood, each heartbeat causes relatively smaller additional pressure changes (flattened waveform) because the system is already taut (23). Additionally, acute SAH is known to cause a surge of catecholamines and potential early vasomotor dysfunction; some arteries may actually dilate or lose tone immediately after hemorrhage (before the development of delayed vasospasm), especially if there is transient hypertension or autoregulatory breakthrough. This vasoparalysis could also lead to a scenario of “vascular collapse” with reduced pulsatile transmission, as described in both experimental and clinical studies linking early autoregulatory failure to blunted arterial pulsations (12, 24, 25).

Interestingly, our observation of initially low pulsatility in SAH animals aligns with a clinical report by Rajajee et al. (6), who found that a low TCD pulsatility index within 48 h of SAH was predictive of subsequent symptomatic vasospasm. They hypothesized that early low PI might reflect a state of relative vasodilation or loss of resistance, preceding the vasospasm constriction phase. In our model, by 24 h the process of vasospasm is just beginning; the PCoA may not yet be severely narrowed (the peak narrowing was at day 7), but the intracranial environment and vascular reactivity are profoundly altered, resulting in diminished pulsations. Thus, the early phase in our SAH rabbits could correspond to that clinical scenario of low PI predicting vasospasm. Indeed, all our SAH animals went on to develop vasospasm by day 7, and all had low pulsation amplitude at day 1—supporting the idea that early pulsatility changes can be a harbinger of impending vasospasm.

By day 7, when vasospasm was fully established in the SAH group (as confirmed by high vasospasm index and histology), the intracranial pulsatility amplitude had increased from its nadir, though not back to normal. Several factors likely contributed to this partial rebound of pulsatility. First, any acute hydrocephalus or markedly raised ICP from the initial hemorrhage would have improved by day 7 as CSF redistributed and the body reabsorbed some subarachnoid blood. A lowering of baseline ICP over time would restore some intracranial compliance, allowing for larger pulse-related volume changes. Second, the vasospasm itself can influence pulsatility: as arteries constrict and become stiffer tubes, the system’s damping capacity changes (6, 24). In systemic arteries, increased stiffness (such as due to atherosclerosis) often leads to *increased pulse pressure* because the elastic buffering is reduced (26). Analogously, a spastic artery might transmit more of the cardiac pulsation into distal cerebral circulation or the CSF space, thereby increasing measured pulsation amplitude. However, severe vasospasm also reduces blood flow, which might be expected to reduce pulsatile volume delivered to the brain each beat (6, 24). The net effect is not intuitive; it could depend on the interplay between reduced flow and increased arterial stiffness. Our results suggest that between day 1 and day 7, the net effect was an increase in pulsation amplitude, implying that the factors of improved compliance and increased stiffness outweighed the factor of reduced flow. By day 7, the pulsation amplitude in SAH animals, though higher than at day 1, was still lower than in the sham-controlled or control groups. We interpret this as an enduring effect of impaired distal perfusion: even though the vessels are stiffer, the amount of blood entering the cranial vault each beat is curtailed by vasospasm, so the pulsations cannot fully normalize.

The PCoA vasospasm index data confirm that the experimental SAH led to robust vasospasm. The index uses morphometric analysis to quantify narrowing. Prior studies by our group and others have used similar measures to evaluate vasospasm severity in experimental models (6, 22, 27). In this study, the sham-controlled group’s moderate increase in VSI (0.99 vs. 0.40 in controls) indicates that even without blood, the mechanical perturbation and possibly transient ICP spike from saline can cause some arterial constriction. This finding is important for experimental design considerations—a true sham-controlled must account for those effects. Nevertheless, the difference between sham-controlled and SAH (0.99 vs. 1.86) groups underscores the specific spasmogenic role of subarachnoid blood. Blood breakdown products such as oxyhemoglobin, iron, nitric oxide and inflammatory mediators are well-known triggers of vasospasm (28–30). Our histological observation of inflammatory infiltrates in spastic arteries supports the contribution of inflammation to vasospasm. The PCoA in SAH animals showed changes (endothelial cell desquamation, elastic lamina changes) that are hallmarks of vasospasm injury and are consistent with other reports of vasospastic cerebral arteries in animal models (28, 29). The integrity of the circle of Willis collaterals is a critical determinant of cerebrovascular resilience after subarachnoid hemorrhage (31). Among these, PCoA is particularly important, as its caliber and patency influence the redistribution of blood flow between anterior and posterior circulations. Clinical studies have demonstrated that a well-developed PCoA can provide substantial protection against ischemia in the setting of ICA occlusion. Conversely, if the PCoA is compromised by vasospasm, this collateral safeguard is effectively lost. Our experimental findings therefore translate into a clinically relevant scenario: a patient with an anterior circulation aneurysm, such as at the ICA terminus, who develops PCoA vasospasm would be at elevated risk for delayed cerebral ischemia due to impaired cross-circulation. This mechanistic link underscores the reviewer’s point that PCoA spasm removes a vital rescue pathway for blood flow, thereby precipitating ischemia in vulnerable territories.

Cerebral autoregulation is frequently impaired in the acute phase of subarachnoid hemorrhage, and this disruption contributes significantly to early hemodynamic instability (32). Blood entry into the subarachnoid space triggers abrupt elevations in intracranial pressure, endothelial dysfunction, and release of vasoactive substances, which collectively blunt the normal vasodilatory and vasoconstrictive responses of cerebral vessels (33). As a result, cerebral blood flow becomes more pressure-dependent, leaving the brain vulnerable to both hypoperfusion and hyperperfusion injury. In our model, the marked reduction in pulsation amplitude during the first 24 h is consistent with this early loss of autoregulatory capacity. Over time, partial recovery of pulsatility was observed by day 7, suggesting that some autoregulatory mechanisms begin to normalize once acute hydrocephalus resolves and the vasospasm phase stabilizes. Clinically, autoregulation is most impaired during the first several days after SAH and often remains disturbed through the peak vasospasm window (days 4–14), with gradual return toward baseline by 2–3 weeks as blood products clear and vascular tone recovers (32, 34). This temporal profile aligns with our findings of depressed but partially improving pulsatility, reinforcing the concept that impaired autoregulation is both an early marker and a modifiable contributor to delayed cerebral ischemia.

An observation in our study is the development of bradycardia in SAH animals by day 7. In clinical SAH, especially when intracranial pressure is elevated, one component of the Cushing reflex is bradycardia in response to hypertension and high ICP (33). While we did not measure systemic blood pressure continuously, the presence of bradycardia suggests that the SAH rabbits may have experienced episodes of increased ICP, particularly in the first days after hemorrhage. By day 7, persistent bradycardia might also result from brainstem ischemia or autonomic dysfunction due to vasospasm near brainstem perforating vessels. The PCoA supplies parts of the midbrain as well as contributes to overall circle perfusion; severe spasm could conceivably compromise those areas. Prior research has shown that SAH can injure autonomic centers and cranial nerve nuclei (11, 33, 35). It is possible that our SAH rabbits had some ischemic insult to the vagal nuclei or other autonomic regulatory regions, leading to a lowered heart rate. The sham-controlled and control groups did not show this change, reinforcing that it is related to the hemorrhage/vessels rather than anesthesia or handling. Clinically, this might parallel how some SAH patients develop neurogenic cardiac changes or bradyarrhythmias.

When considering clinical implications, our findings highlight cerebral pulsatility as a potential monitoring parameter in SAH. Currently, clinicians focus on flow velocities (via TCD) and clinical exam to infer vasospasm. However, pulsatility index changes may provide additional clues. For instance, a trend of initially low PI that later rises could indicate a transition from hyperdynamic/low-resistance state to a high-resistance vasospastic state. Some authors have noted that increased PI during vasospasm could reflect distal small-vessel narrowing that might not markedly elevate proximal velocities (34, 36). Our data support this: by day 7, despite severe vasospasm, our model’s “pulsatility” was increased relative to day 1, though still depressed relative to normal, suggesting a complex interplay. If translated, one might expect a patient’s ICP pulse amplitude or TCD PI to dip right after hemorrhage (due to global acute effects) and then climb as focal vasospasm sets in. Continuous monitoring of ICP pulsatility in neurocritical care (e.g., using an external ventricular drain or noninvasive methods) could potentially serve as an early warning for vasospasm or ineffective collateral flow. This concept is analogous to how systemic arterial stiffness is monitored via pulse pressure; here intracranial arterial “stiffness” from vasospasm could be tracked via ICP pulse amplitude.

Another implication is that treatments aimed at relieving vasospasm (such as vasodilator infusions or angioplasty in patients) might be evaluated by noting improvements in pulsatility. In theory, alleviating vasospasm should restore a more normal pulsatile flow pattern (25, 37). In current clinical practice, intra-arterial administration of vasodilators such as verapamil, nicardipine, or milrinone is frequently employed for the endovascular treatment of severe cerebral vasospasm. These interventions can improve vessel caliber and sometimes neurological status, but clinical trial data emphasize that their long-term impact on outcomes remains uncertain (38, 39). Future research could test whether therapies (e.g., intraarterial nimodipine or verapamil) in models like ours improve both vessel caliber and pulsatility measures.

The causal link between PCoA vasospasm and impaired intracranial pulsatility warrants careful interpretation. In our model, the sequence of events supports vasospasm as the primary driver of pulsatility changes. The initial insult precipitates vasospasm, and a marked reduction in pulsation amplitude was already evident at 24 h, preceding the establishment of chronic vasospasm by day 7. By this later stage, histological analysis confirmed severe vasospasm, and pulsatility remained significantly depressed. This timeline is consistent with the understanding that cerebral vasospasm is triggered by blood breakdown products, oxidative stress, and inflammatory mediators following SAH, rather than by alterations in pulsatile flow per se (29, 40). Moreover, an exploratory analysis revealed an inverse trend between vasospasm severity and pulsation amplitude (Spearman *ρ* = −0.45, *p* = 0.012), suggesting that greater arterial narrowing is associated with stronger dampening of pulsatility. Mechanistically, vasospastic arteries increase local vascular resistance and stiffness, which can distort or attenuate the transmission of arterial pulse waves into the cranial cavity, thereby lowering ICP pulse amplitude. Conversely, there is no evidence that a reduction in pulsation amplitude alone can induce focal arterial spasm. While we cannot completely exclude complex bidirectional interactions, the most parsimonious interpretation is that SAH-induced PCoA vasospasm impairs cerebral pulsatility, rather than pulsatility abnormalities provoking vasospasm. We therefore acknowledge alternative explanations but emphasize that the temporal sequence, the inverse correlation, and established pathophysiology all favor vasospasm as the causal factor.

Our findings also have important translational implications for anterior circulation aneurysms and the development of delayed cerebral ischemia (41). The majority of aneurysmal SAH cases occur in the anterior circulation, including aneurysms of the anterior communicating artery, middle cerebral artery, and the internal carotid artery near the PCoA origin (42). Even in these situations, the posterior communicating artery plays a critical collateral role. For example, if the internal carotid artery is compromised by aneurysmal rupture or during surgical/endovascular treatment, the PCoA can redirect flow from the posterior to the anterior circulation. Vasospasm of the PCoA in such cases would abolish this collateral function and could significantly worsen outcomes (43).

An additional consideration in the interpretation of our findings relates to the potential influence of sex hormones on the pathophysiology of SAH and vasospasm. All rabbits used in the present study were male New Zealand White rabbits. While this ensured experimental uniformity, it also limits generalizability, as hormonal status is known to influence cerebrovascular reactivity (44, 45). Estrogen, in particular, has been shown to exert vasoprotective effects by promoting endothelial nitric oxide production, maintaining vascular elasticity, and attenuating inflammatory responses (46). Clinical data indicate that premenopausal women, with higher circulating estrogen levels, may have partial protection against aneurysm formation and possibly reduced vasospasm severity, whereas postmenopausal women face greater risk due to estrogen deficiency (47).

It is important to acknowledge the limitations of this study. First, the sample size is modest, especially effectively 13 in the SAH group after attrition. While sufficient to show clear differences, a larger cohort would allow more granular analysis, such as correlation between degree of vasospasm and neurological deficits or mortality. Second, our measurement of cerebral pulsatility was indirect. We used ICP pulse amplitude as a surrogate for cerebral blood flow pulsatility. Although related, the relationship between ICP pulsations and cerebral blood flow pulsations can be affected by many factors, including intracranial compliance and venous outflow. Ideally, we would complement this with direct CBF measurements (e.g., TCD of middle cerebral artery in rabbits or laser Doppler cerebral blood flow). However, reliable TCD insonation in a small animal is technically challenging. Our approach captured the global intracranial consequence of altered flow dynamics. Third, the focus on the PCoA, while novel, means we did not directly assess other arteries like the middle cerebral or basilar artery. It is likely that vasospasm was not limited to the PCoA in our SAH model—other arteries in the circle of Willis probably also went into spasm. We singled out PCoA because of our interest in its role and because its small size allowed a sensitive measure of vasospasm. This could limit direct generalization to larger arteries; the absolute pulsatility effect might differ if a major vessel like MCA were spastic. Nonetheless, PCoA spasm is clinically relevant, particularly for its collateral function, and our model simulates a scenario of compromised collateral routes.

Another limitation is that our physiological measurements were done under anesthesia, which depresses overall neuronal activity and can alter vascular tone (e.g., xylazine has hypotensive and bradycardic effects via α2-agonism). We attempted to minimize this by using consistent anesthetic protocols across groups and time points. Any residual anesthetic effect would equally affect control, sham-controlled, and SAH at each measurement, so the relative differences should remain valid. Still, absolute values like heart rate were certainly influenced by anesthesia (rabbits have higher HR when awake). In future studies, using telemetry to record pulsations and heart rate in conscious freely moving animals would be ideal, though technically complex.

Despite these limitations, the internal consistency of our data (e.g., all SAH animals showed the expected trends, all controls remained stable) strengthens confidence in the conclusions. The coupling of physiological data with histological confirmation is a particular strength. We clearly demonstrated that the animals with the greatest pulsatility disturbances were the ones with severe anatomically confirmed vasospasm. This cause-effect link is bolstered by the sham-controlled group evidence that without blood, severe vasospasm did not occur and pulsatility remained normal.

In the context of previous research, our study adds a new dimension to understanding SAH outcomes. Classical research has primarily concerned itself with absolute cerebral blood flow reductions and tissue infarction as endpoints of vasospasm. However, more recent attention has been paid to “early brain injury” immediately after SAH and to the role of microcirculatory dysfunction and cortical spreading depolarizations in delayed ischemia (24). Pulsatility changes could be a marker of microvascular dysfunction; a stiff microcirculation might reflect loss of autoregulation. There is some evidence that a high pulsatility index correlates with poor outcomes in stroke and traumatic brain injury patients by indicating poor distal compliance (21–23). In our experiment, even though we did not directly measure outcome beyond 1 week, one could speculate that the persistent pulsatility impairment in the SAH group might correlate with worse brain perfusion or ongoing risk of ischemia had the animals lived longer. It would be informative in future studies to assess cognitive or behavioral outcomes and correlate them with the degree of pulsatility restoration or impairment.

Finally, our results also highlight that even mild vasospasm (as in the sham-controlled group) can occur from procedures and should be accounted for. Researchers using intracisternal injections in animals should be aware that the vehicle (saline) is not entirely inert; it can induce a mild inflammatory or mechanical vasospasm. In our sham-controlled animals, however, this mild vasospasm did not significantly affect intracranial pulsatility or cause neurological deficits, suggesting there is a threshold of vasospasm severity below which global pulsatile hemodynamics remain intact. This threshold concept might apply clinically: minor angiographic vasospasm might not impact patients’ hemodynamics or symptoms, whereas severe vasospasm does.

## Conclusion

In summary, we found that posterior communicating artery vasospasm leads to significant disturbances in cerebral pulsatility in a rabbit model of subarachnoid hemorrhage. The vasospasm was associated with an initial suppression of intracranial pressure pulsations followed by an incomplete recovery, indicating that normal pulsatile flow dynamics were impaired. These hemodynamic changes paralleled the severity of PCoA narrowing observed histologically. Our study underscores the importance of considering not only gross cerebral blood flow reduction but also the qualitative changes in blood flow pulsatility as a consequence of vasospasm. Monitoring cerebral pulsatility (for example, via TCD-derived PI or direct ICP pulse monitoring) may provide additional insight into the cerebrovascular state of SAH patients, potentially aiding in the early detection of dangerous vasospasm or guiding therapeutic interventions. Further research is warranted to explore pulsatility as a diagnostic and prognostic tool in SAH and to determine if therapies that mitigate vasospasm can also normalize cerebral pulsatility, thereby improving patient outcomes.

## Data Availability

The raw data supporting the conclusions of this article will be made available by the authors, without undue reservation.
