# Supplementary material for: Posterior communicating artery vasospasm impairs cerebral pulsatility in an experimental subarachnoid hemorrhage model
Source: Front Neurol. 2025 Oct 8;16:1649577. doi: 10.3389/fneur.2025.1649577 (PMC12540087; doi:10.3389/fneur.2025.1649577)
Supplement: Supplementary file 1 [file Table_1.docx]

**Supplementary Table 1. Individual Vasospasm Index (VSI) Values for Experimental Animals**

| **Animal ID** | **Group** | **VSI Value** |
| --- | --- | --- |
| C1 | Control | 0.42 |
| C2 | Control | 0.33 |
| C3 | Control | 0.45 |
| C4 | Control | 0.47 |
| C5 | Control | 0.26 |
| S1 | Sham | 0.86 |
| S2 | Sham | 1.00 |
| S3 | Sham | 0.96 |
| S4 | Sham | 0.99 |
| S5 | Sham | 0.90 |
| SAH1 | SAH | 2.04 |
| SAH2 | SAH | 2.02 |
| SAH3 | SAH | 1.87 |
| SAH4 | SAH | 2.10 |
| SAH5 | SAH | 1.96 |
| SAH6 | SAH | 1.68 |
| SAH7 | SAH | 1.94 |
| SAH8 | SAH | 1.66 |
| SAH9 | SAH | 2.04 |
| SAH10 | SAH | 1.85 |
| SAH11 | SAH | 1.82 |
| SAH12 | SAH | 1.72 |
| SAH13 | SAH | 2.12 |
